# Supplementary material for: Most researchers would receive more recognition if assessed by article-level metrics than by journal-level metrics
Source: PLoS Biol. 2025 Dec 16;23(12):e3003532. doi: 10.1371/journal.pbio.3003532 (PMC12707641; doi:10.1371/journal.pbio.3003532)
Supplement: S1 Text — Table A: Binomial test on race: We conducted 2-sided binomial test and used binom_test function in the stats module from SciPy library and Python 3.8.8 version for the binomial test. All 4 racial categories showed significance across all three thresholds (impact factor ≥ 10, 15, or 20, respectively). Table B: Chi-squared test on gender (all 3 thresholds showed significance): We used Python’s stats module from SciPy library and python 3.8.8 version for the Chi-squared test. Table C: Chi-squared test on race: We used Python’s stats module from SciPy library and Python 3.8.8 version for the Chi-squared test. We classified each scientist in a particular race if the probability of the researcher being in that racial group is greater than 0.50. Table D: Paired sample t test on Citation Elites versus Journal Elites based on career stage: We conducted a matched-pairs t test for each career stage across all the thresholds. We used Python’s stats module from the SciPy library and the Python 3.8.8 version for the test. We define early career researchers as those who have a career age of 5 years or less, and mid-career researchers as those who have a career age of 10 years or less but more than five. The rest are categorized as senior researchers. Table E. Investigators stratified by race whose papers are most frequently recognized in the Journal Elite versus Citation Elite categories. Chi-squared table for race at an impact factor threshold of 15. Chi-squared: 50.026, p-value: 7.887060421743808e-11. Table F. Investigators stratified by gender whose papers are most frequently recognized in the Journal Elite versus Citation Elite categories. Chi-squared: 40.575, p-value: 1.8922670956226282e-10′. Table G: Investigators stratified by career stage whose papers are most frequently recognized in the Journal Elite versus Citation Elite categories. Chi-squared: 8.289, p-value: 0.0158. Table H: KS Test on Racial Probability: We conducted 2-sided k-s test on each racial probability score [file pbio.3003532.s001.docx]

# Supplemental Materials

## Statistical Analysis

| **Threshold** | **Race** | **P value** |
| --- | --- | --- |
|  | Asian | < 0.0001 |
| Threshold 10 | Black | < 0.0001 |
|  | White | < 0.0001 |
|  | Hispanic | < 0.0001 |
|  | Asian | < 0.0001 |
| Threshold 15 | Black | < 0.0001 |
|  | White | < 0.0001 |
|  | Hispanic | < 0.0001 |
|  | Asian | < 0.0001 |
| Threshold 20 | Black | < 0.0001 |
|  | White | < 0.0001 |
|  | Hispanic | < 0.0001 |

**Table A**: Binomial test on race: We conducted 2-sided binomial test and used binom_test function in the stats module from scipy library and python 3.8.8 version for the binomial test. All 4 racial categories showed significance across all three thresholds (impact factor >= 10, 15, or 20, respectively).

| Threshold | chi sq statistic | p-value |
| --- | --- | --- |
| 10 | 61.77 | 3.842834850588465e-15 |
| 15 | 40.57 | 1.8922670956226282e-10 |
| 20 | 9.87 | 0.0016 |

**Table B**: Chi sq test on gender (all 3 thresholds showed significance): We used python’s stats module from scipy library and python 3.8.8 version for the Chi-square test.

| Threshold | chi sq statistic | p-value |
| --- | --- | --- |
| 10 | 96.81 | 7.510471124857204e-21 |
| 15 | 50.026 | 7.887060421743808e-11 |
| 20 | 30.8199 | 9.275798428124219e-07 |

**Table C**: Chi-square test on race: We used python’s stats module from scipy library and python 3.8.8 version for the Chi-square test. We classified each scientist in a particular race if the probability of the researcher being in that racial group is greater than 0.50.

| Career Stage | P-value |
| --- | --- |
| Early | 9.049375221567279e-75 |
| Mid | 0.0 |
| Senior | 0.0 |

**Table D**: Paired sample t-test on Citation Elites vs Journal Elites based on career stage: We conducted a matched-pair t-test for each career stage across all the thresholds. We used python’s stats module from the scipy library and the Python 3.8.8 version for the test. We define early career researchers as those who have a career age of five years or less and mid-career researchers as those who have a career age of ten years or less but more than five. The rest are categorized as senior researchers.

|  | Asian | Black | White | Hispanic |
| --- | --- | --- | --- | --- |
| Journal Elite | 495 | 16 | 1687 | 65 |
| Citation Elite | 5244 | 319 | 25713 | 1051 |

**Table E**. Investigators stratified by race whose papers are most frequently recognized in the Journal Elite vs. Citation Elite categories. Chi sq table for race at an impact factor threshold of 15. Chi-sq: 50.026, p value: 7.887060421743808e-11

|  | Female | Male |
| --- | --- | --- |
| Journal Elite | 592 | 1351 |
| Citation Elite | 10909 | 18017 |

**Table F**. Investigators stratified by gender whose papers are most frequently recognized in the Journal Elite vs. Citation Elite categories. Chi sq: 40.575, p value: 1.8922670956226282e-10

|  | Early | Mid | Senior |
| --- | --- | --- | --- |
| Journal Elite | 71 | 201 | 2026 |
| Citation Elite | 717 | 2813 | 29341 |

**Table G**: Investigators stratified by career stage whose papers are most frequently recognized in the Journal Elite vs. Citation Elite categories. Chi-sq: 8.289, p-value: 0.0158.

| Threshold (Impact Factor 15+) | p-value |
| --- | --- |
| Asian | < 2.2e-16 |
| Black | < 2.2e-16 |
| Hispanic | < 2.2e-16 |
| White | < 2.2e-16 |

**Table H:** KS Test on Racial Probability:
We conducted 2-sided k-s test on each racial probability score for scientists who receive more recognition under article vs journal-level metrics (also see Fig A). The p-value of the k-s test is significant across all three thresholds (impact factor >= 10, 15, or 20, respectively).

| 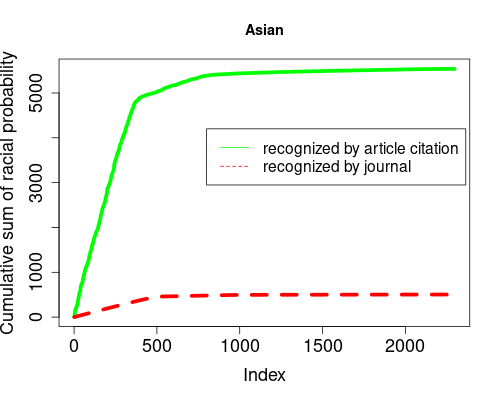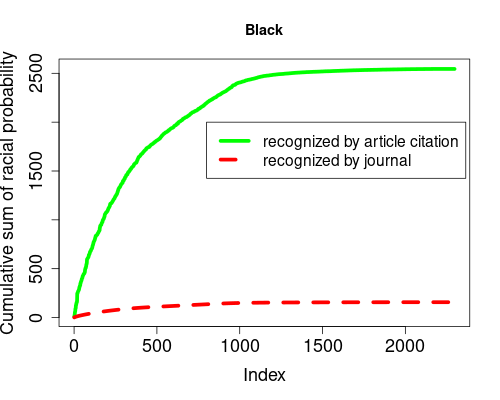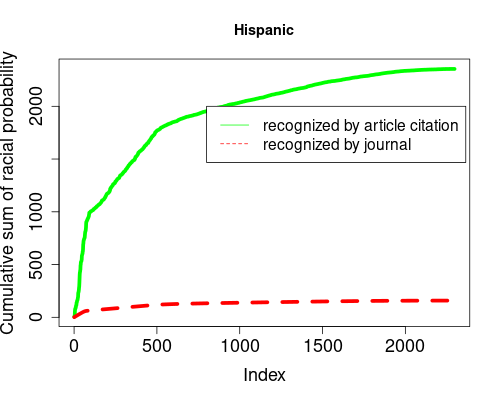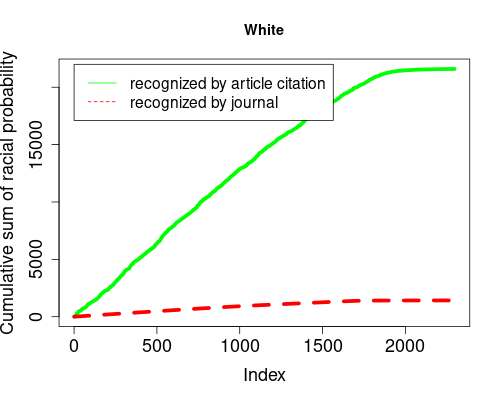 |
| --- |
| **Fig A**: Cumulative probability distribution of racial score of scientists who are more recognized as Citation Elites vs. Journal Elites. p-values shown in Table H. |

| **Race** | **p-value** |
| --- | --- |
| Asian | < 0.0001 |
| Black | 0.19 |
| White | < 0.0001 |
| Hispanic | 0.33 |

**Table I**: Proportion-z test on race: We conducted 2-sided proportion z test across all racial categories using proportions_ztest function in the stats module from statsmodels library and python 3.8.8 version for the test. We get similar significance across all thresholds.

**All racial groups benefit from article-level metrics.** Many scientists would receive more recognition under the article-level evaluation metric and this trend persists across all the racial groups. We conducted a 2-sided k-s test on each racial probability score for scientists who receive more recognition under article-level RCR vs journal-level metrics. The p-value of the k-s test is significant across all three thresholds (impact factor >= 10, 15, or 20, respectively). We also conducted 2-sided binomial tests on each racial group for both article-level RCR and article citation rate. The p-value of the test is significant across ass three thresholds (impact factor >= 10, 15, or 20, respectively) suggesting that a higher proportion of people will benefit from ALM for each racial group.

Though our analysis suggests that each racial group will get more recognition under the ALM, this magnitude of benefit could be different for different racial groups (Table S9). For Blacks and Hispanics, there is no significant difference in the proportion of the particular racial group in the citation elite vs journal elite group across different thresholds (impact factor >= 10, 15, 20) for both article-level metrics - RCR and article-citation rate. For Asians, the proportion of Asians in the citation elite is significantly lower than the proportion of Asians in the journal elite group across different thresholds (impact factor >= 10, 15, 20) for RCR. For whites, the proportion of whites in the citation elite is significantly higher than the proportion of whites in the journal elite group for all thresholds (10, 15, 20) for RCR. Similar results were seen with article citation rate that does not include field normalization.

| 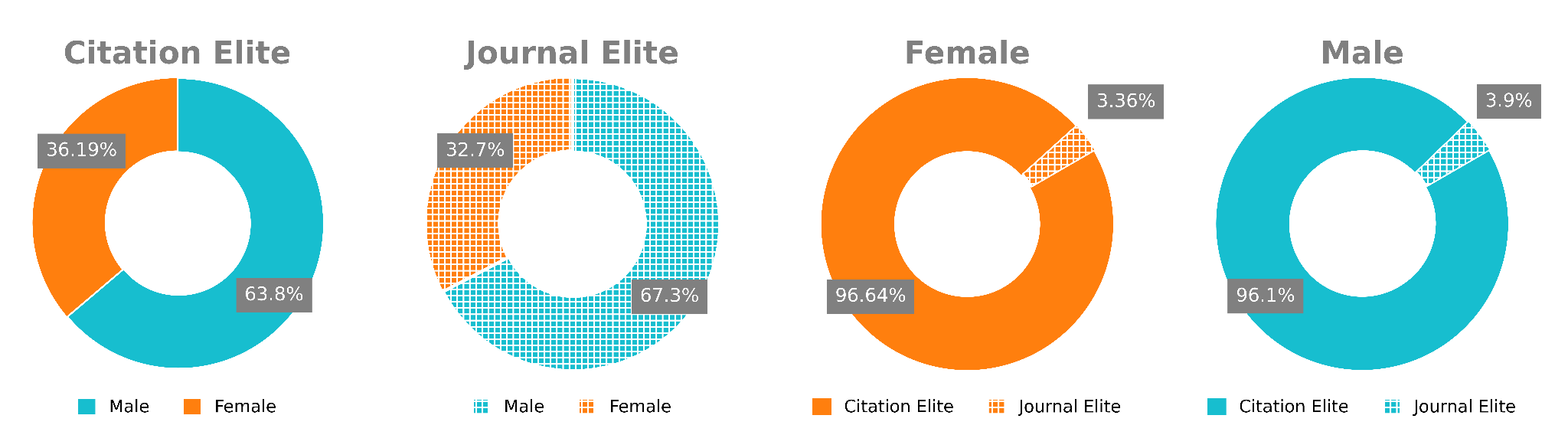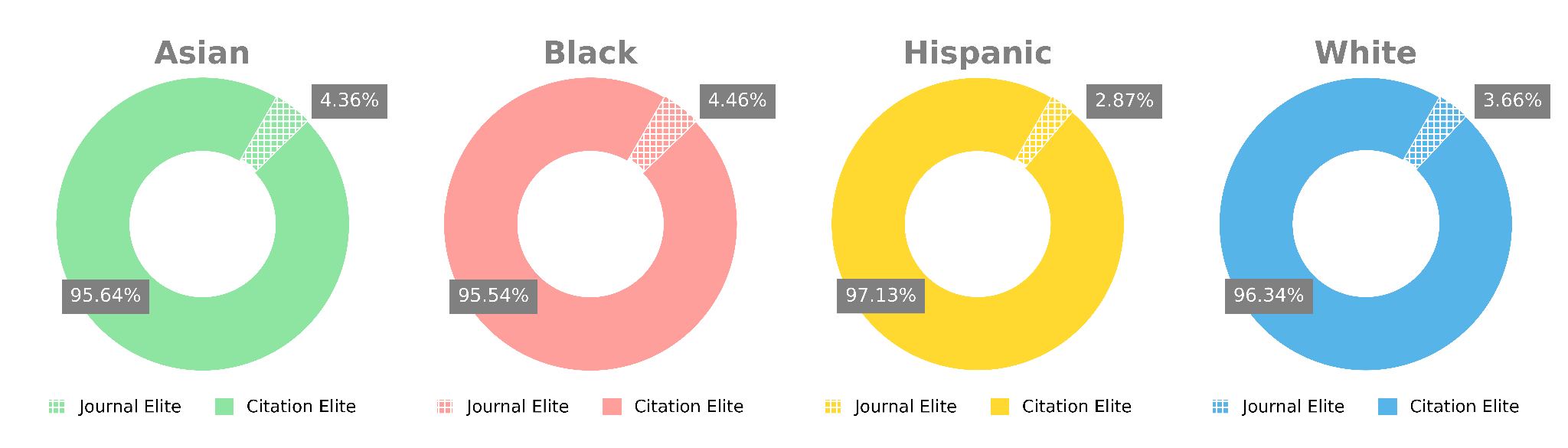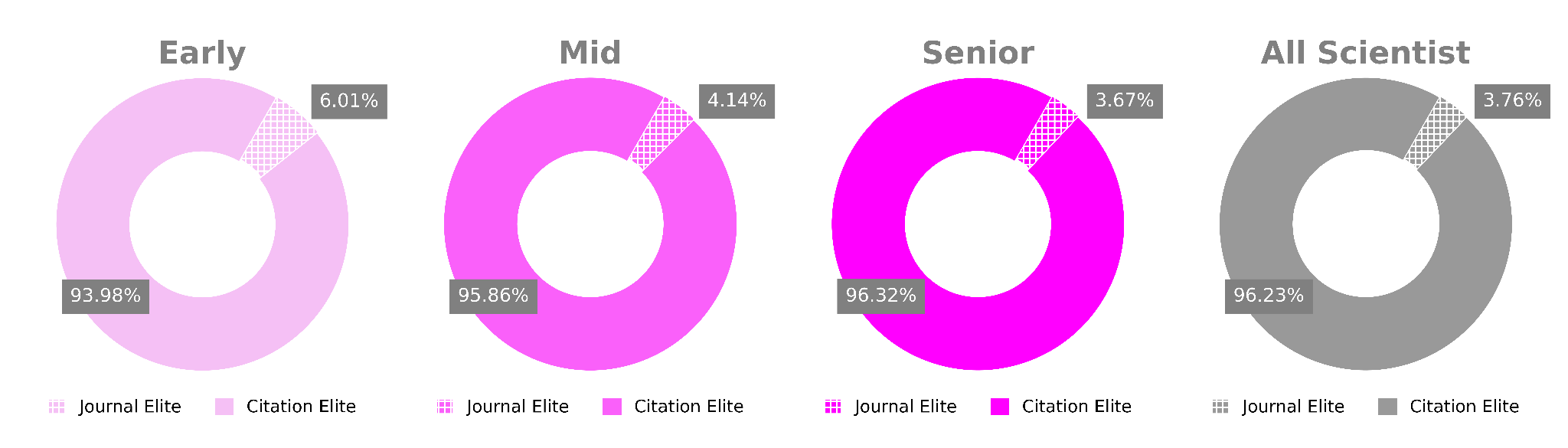 |
| --- |
| **Fig B**: Demographic analysis of scientists who receive more recognition with journal- vs. article-level measures (Article-citation rate). (Top, within box) Breakdown of how many scientists filtered by those who have more Citation Elite (these scientists are labeled as Citation Elites) papers than Journal Elite papers (labeled as Journal Elites) are male vs. female. (Top, outside box) Breakdown of how many scientists filtered by gender receive more recognition with article-level citations (Citation Elite) vs. journal impact factor (Journal Elite). (Middle) Breakdown filtered by race. (Bottom) Breakdown filtered by seniority. |

### Correlation Analysis

We analyzed the correlation between the metric and publication per year. We observe a weak but significant correlation between article-level metric and the publication per year. The Pearson correlation coefficient between article-level metric and publication per year is 0.08 with a p-value < 0.001. However, there is no significant correlation between journal-level metric and publication per year (Pearson correlation coefficient 0.008, p-value = 0.058).

### Subgroup Analysis

| Category | Metric | male female |
| --- | --- | --- |
| Top 10k profile (ALM) | Citation Elite  Journal Elite | 64.33 35.67  69.44 30.54 |
| Top 10k profile (JIF) | Citation Elite  Journal Elite | 70.55 29.45  69.38 30.61 |
| Bottom 10k profile (JIF) | Citation Elite  Journal Elite | 69.12 30.33  70.19 28.85 |

**Table J:** Percentage of male and female scientists in citation elite and journal elite.

| Category | Gender Group | Citation Elite Journal Elite |
| --- | --- | --- |
| Top 10k profile (ALM) | Female  Male | 97.41 2.589  96.76 3.24 |
| Top 10k profile (JIF) | Female  Male | 76.53 23.47  77.51 22.49 |
| Bottom 10k profile (JIF) | Female  Male | 98.77 1.22  98.67 1.33 |

**Table K:** Percentage of citation elite and journal elite across gender groups.

| Category | Racial Group | Citation Elite Journal Elite |
| --- | --- | --- |
| Top 10k profile (ALM) | Asian  Black  White  Hispanic | 95.06 4.94  96.15 3.85  97.07 2.93  97.40 2.59 |
| Top 10k profile (JIF) | Asian  Black  White  Hispanic | 72.27 27.72  81.36 18.64  77.70 22.29  77.64 22.36 |
| Bottom 10k profile (JIF) | Asian  Black  White  Hispanic | 98.55 1.45  98.91 1.09  98.69 1.31  99.14 0.855 |

**Table L:** Percentage of citation elite and journal elite across racial groups.

| Category | Career Group | Citation Elite Journal Elite |
| --- | --- | --- |
| Top 10k profile (ALM) | Early  Mid  Senior | 96.43 3.56  97.13 2.86  96.75 3.25 |
| Top 10k profile (JIF) | Early  Mid  Senior | 56.79 43.21  72.67 27.32  77.51 22.49 |
| Bottom 10k profile (JIF) | Early  Mid  Senior | 100 0.00  98.73 1.27  98.66 1.34 |

**Table M:** Percentage of citation elite and journal elite across career groups.

| Category | Citation Elite | Journal Elite |
| --- | --- | --- |
| Top 10k profile (ALM) | 96.78 | 3.22 |
| Top 10k profile (JIF) | 76.69 | 23.31 |
| Bottom 10k profile (JIF) | 98.66 | 1.3 |

**Table N:** Percentage of citation elite and journal elite across the entire dataset.

### Threshold 15

| Test | Hits | Acr |
| --- | --- | --- |
| Binomial test | Asian (hits greater - sig) - 2-sided p (< 0.0001)  Black (hits greater - sig) - 2-sided p (< 0.0001)  White (hits greater - sig) - 2-sided p (< 0.0001)  Hispanic (hits greater - sig) - 2-sided p (< 0.0001) | Asian (hits greater - sig) - 2-sided p (< 0.0001)  Black (hits greater - sig) - 2-sided p (< 0.0001)  White (hits greater - sig) - 2-sided p (< 0.0001)  Hispanic (hits greater - sig) - 2-sided p (< 0.0001) |
| Ks test | Asian (hits greater - sig) - 2-sided p (< 0.0001)  Black (hits greater - sig) - 2-sided p (< 0.0001)  White (hits greater - sig) - 2-sided p (< 0.0001)  Hispanic (hits greater - sig) - 2-sided p (< 0.0001) | Asian (hits greater - sig) - 2-sided p (< 0.0001)  Black (hits greater - sig) - 2-sided p (< 0.0001)  White (hits greater - sig) - 2-sided p (< 0.0001)  Hispanic (hits greater - sig) - 2-sided p (< 0.0001) |

**Table O:** Statistical tests on threshold 15.

### Threshold 10

| Test | Hits | Acr |
| --- | --- | --- |
| Binomial test | Asian (hits greater - sig) - 2-sided p (< 0.0001)  Black (hits greater - sig) - 2-sided p (< 0.0001)  White (hits greater - sig) - 2-sided p (< 0.0001)  Hispanic (hits greater - sig) - 2-sided p (< 0.0001) | Asian (hits greater - sig) - 2-sided p (< 0.0001)  Black (hits greater - sig) - 2-sided p (< 0.0001)  White (hits greater - sig) - 2-sided p (< 0.0001)  Hispanic (hits greater - sig) - 2-sided p (< 0.0001) |
| Ks test | Asian (hits greater - sig) - 2-sided p (< 0.0001)  Black (hits greater - sig) - 2-sided p (< 0.0001)  White (hits greater - sig) - 2-sided p (< 0.0001)  Hispanic (hits greater - sig) - 2-sided p (< 0.0001) | Asian (hits greater - sig) - 2-sided p (< 0.0001)  Black (hits greater - sig) - 2-sided p (< 0.0001)  White (hits greater - sig) - 2-sided p (< 0.0001)  Hispanic (hits greater - sig) - 2-sided p (< 0.0001) |

**Table P:** Statistical tests on threshold 10.

### Threshold 20

| Test | Hits | Acr |
| --- | --- | --- |
| Binomial test | Asian (hits greater - sig) - 2-sided p (< 0.0001)  Black (hits greater - sig) - 2-sided p (< 0.0001)  White (hits greater - sig) - 2-sided p (< 0.0001)  Hispanic (hits greater - sig) - 2-sided p (< 0.0001) | Asian (hits greater - sig) - 2-sided p (< 0.0001)  Black (hits greater - sig) - 2-sided p (< 0.0001)  White (hits greater - sig) - 2-sided p (< 0.0001)  Hispanic (hits greater - sig) - 2-sided p (< 0.0001) |
| Ks test | Asian (hits greater - sig) - 2-sided p (< 0.0001)  Black (hits greater - sig) - 2-sided p (< 0.0001)  White (hits greater - sig) - 2-sided p (< 0.0001)  Hispanic (hits greater - sig) - 2-sided p (< 0.0001) | Asian (hits greater - sig) - 2-sided p (< 0.0001)  Black (hits greater - sig) - 2-sided p (< 0.0001)  White (hits greater - sig) - 2-sided p (< 0.0001)  Hispanic (hits greater - sig) - 2-sided p (< 0.0001) |

**Table Q:** Statistical tests on threshold 20.
