# Supplementary material for: Most researchers would receive more recognition if assessed by article-level metrics than by journal-level metrics
Source: PLoS Biol. 2025 Dec 16;23(12):e3003532. doi: 10.1371/journal.pbio.3003532 (PMC12707641; doi:10.1371/journal.pbio.3003532)
Supplement: Attachment — Submitted filename: REVIEWERS_R2R_20250905.docx [file pbio.3003532.s010.docx]

REVIEWERS' COMMENTS:


Reviewer #1:

The article by Arabi et colleagues performs a bibliometric analysis of citation impact that contrasts a journal-level readout against an article-level readout. They arrive at the findings that the former overlooks influential science, whereas the latter results in a more equitable recognition of scientists.

The main advance of this work lies in providing more data-driven insights when exploring these  two classes of readouts. Building on top of this advance, they also discover that the scenario (article-level) that appears farther from the current reality promises to simultaneously allow for a more individualistic (pieces of works) and equitable (better representation of diverse scientists) recognition of scientific influence. I greatly enjoyed the basic idea of using data to compare article-level versus journal-level metrics, and personally believe that any resulting findings would be helpful (even absence of differences) to further ground ongoing debates on how to improve science. Further adding to the appeal, their specific findings already imply an actionable strategy on how to yield a more equitable science, and thus could be equally helpful to scientists, organizations, and policy makers. However, several methodological points, and the framing of the manuscript,
regretfully must dampen my enthusiasm as they could undermine multiple of the manuscript's conclusions. I hope that below comments will strengthen the manuscript rather than discourage the authors from pursuing their valuable line of research.

*- Thank you for the kind words and enthusiastic invitation for an improved revision.*

My first set of methodological remarks relate to absolute numbers, and usage of membership to citation-elite as a metric.

One of the central ideas of the manuscript is that shifting from a journal-level metric to an article-level metric could promote more equitable decisions (particularly around hiring/promotion) and lead to a more complete recognition of influential science. However, these conclusions do not consider absolute numbers in publications and available slots for hiring/promotion.

Besides citations, the absolute number of publications is informative of hiring/promotion, as quantitatively demonstrated by Dijk et al., Current Biology, 2014. However, this is not controlled for. Nor do they consider additional complexities, such as the varying time investments for publication in different journal tiers.

*- Yes, we agree that the number of publications is certainly a consideration. In this study, the number of publications was controlled for by fixing the number/identities of papers for each author, so that on an author level, the journal-level metrics have the same number of publications as the article-level metrics. We have also added discussion about this consideration into the manuscript related to the Dijk et al. paper.*

The number of available slots for hiring/recruiting is finite. Based on the description of the authors, they partially to address this "zero-sums framework" by investigating whether authors gain some recognition - meaning that they either published as part of the "Journal Elite" or "Citation Elite". However, the actual number of slots could be more finite, meaning that rather than asking whether authors have one paper of the citation elite, hiring/recruiting committees would need to modulate their search heuristic. Would the authors findings' on equity change, if one were to - again - limit to the very top individuals within one elite (e.g.: those with highest share of citation elite papers, or those with most citation elite papers, ….)?

*- We appreciate the thoughtful suggestion. We agree that the number of positions is likely to exceed the number of applicants. The main contribution here is to quantitatively demonstrate the disparity as to who may be given consideration in an exclusively journal-based system (where again, the number of publications is fixed) vs when article-level metrics are taken into consideration. We have also added a subgroup analysis to the supplement, focusing on the top authors as suggested.*

The authors imply that membership in the citation elite could be a better metric for hiring/recruiting decisions, or an improvement of the recognition of a large segment of the biomedical workforce. However, the authors do not investigate whether article-level metrics are better in discriminating between authors of future influential science than journal-level metrics (this may even be difficult to explore given the possibility for confounding factors).

*- We agree about potential confounds, and consider this to be out of scope for the present analysis, but a promising future direction.*

Related to above paragraph, I am not convinced that the specific flavor of article-level metrics sketched by the authors (having at least one paper in citation elite) is the most appropriate article-level metric. Measures of citations typically show some distributions - and this also holds for authors for the citation metric (RCR) used in this study, as shown in their earlier publication Hutchins et al. 2016. Thus, the presence of at least one publication within the citation elite should scale (though not necessarily linearly) with the total number of publications published. The pronounced lines in the scatter plots of Figure 2 and 6 suggest that most authors only have few papers as part of the citation elite. Some of the membership to the citation elite may be spurious or an indirect indicator of the total number of papers published, rather than a qualitative difference in the published work, when compared to the papers of other authors.

*- This is a valid concern. We have quantified this correlation. There is a statistically significant correlation between the fraction of papers that are citation elite and the number of publications, but it is weak (Pearson r = 0.08). We have reported this in our revision. Further, the fraction of authors with fewer than 10 publications in their profile is low (less than 10%). Further discussion of these points has been added to the Results regarding the Figure 2 data.*

Further adding to my concern, the
authors conclusions - on Figures 3 and 5 related to equity - on article-level metrics better approximating the overall composition of the workforce should all also hold true if their metric was performing randomly or overly inclusive (as suggested by order of magnitude difference in authors; also see above point on finite slots for hiring/promotion).
*- We agree that the ~10-fold difference is indeed large and of great practical significance. However, we believe our zero-sum analysis, which shows that approximately half of authors lack a journal elite paper, while approximately 25% of authors lack a citation elite paper, does not constitute an overly inclusive measure.*

How many of the authors of the citations elite are significantly more elite when compared to the entirety of the considered workforce? How would findings on equity change if only considering significant authors, or further filtering the significant authors for the top 1,000 or 10,000 most significant authors?

*- As mentioned above, we have added this subgroup analysis to our supplement with similar results.*

Related to above points, but exceeding the specific comments, the present work follows a narrative of publications in journal elites being drivers of major career decisions. This is also used as opening sentence to introduce the need for the current study. Though this may be a common narrative, the authors do not provide data to back this assumption. Nor do they cite publications with corresponding data (only opinion articles). Inversely, I do not believe that this narrative generalizes. For instance, once a member of the National Academy of Science shared with me that, in her view, many junior biomedical investigators made the mistake of believing that the pursuit of publications in Nature, Science, Cell was the ideal strategy. However, hiring committees would more favorably look upon candidates that instead invested their fine time to publish multiple papers in specialist journals. The reason for this search heuristic would be that constant productivity in one domain was
anticipate to increase the prospects for obtaining future federal grants. While some of these facets might be tricky to address in a quantitative study without further surveys, Dijk et al., Current Biology, 2014 similarly already demonstrated that hiring correlates with multiple quantifiable metrics beyond citations alone (including reputation of school, number publications). Either suggests that a more nuance in the framing of the manuscript and its discussion would be required.
*- We agree that these other factors are indeed important and have added additional context to the Discussion along these lines. The advice from the NAS member mentioned above seems sound. We limit our comparison to the citation-based metrics (journal vs article) so that the results are easily interpreted in this context.*

My second set of methodological remarks relate to the way that citations themselves are measured and presented.

Arabi et colleagues use a high citation metric (specifically, RCR metric) as an indicator of citation elite, and argue that many of these manuscripts are missed by only looking at high impact factor journals. While this statement is true, the authors further heavily imply that this is lack of recognition and influence, or articles could be overlooked by research assessment - even speaking of "misallocation".
*- We have toned down this language.*

However, the data points most visible in Figure 1 seem to counter such a narrative. Specifically, I spent 10 minutes to identify the 10 papers with the highest RCR (after 2010) in mBio, Yale J Biol Med, and World J Surg, to see what those papers are, and if there would be well-identifiable differences to papers published by Science, Nature, Cell, and hence help to better interpret the work of Arabi et al.

In mBio, a microbiology journal, 6 relate to SARS-CoV (including one paper from 2018 on SARS-CoV-1 instead of SARS-CoV-2). As all of them have >=70 citations per year (much more than the cutoff used by Arabi et al. for high-impact journals) this suggests that the scientific community can recognize influential publications (irrespective of RCR). Simultaneously, the example of SARS-CoV shows that factors other than the inherent quality of scientific works can influence citations (such as timeliness to a pandemic, a probabilistic global event). Further, that influential work on SARS-CoV could get recognized even if not published in Science, Nature, Cell.

In Yale J Biol Med, 10 of the 10 top ranked citation elite manuscripts are review articles. This could be seen as a motivation for an analysis stratified by article types, as review articles could yield more citations than primary research. However, this also undermines part of the jump of the authors toward a lack recognition, as review articles on specialist topics could be seen as one form of recognition and influence. More generally, this observation could lessen the findings as 30% of the papers with an RCR >3.4 are not research articles (if we stick to the definition of research articles as used in iCite), which is an increase over the 19% among all papers (and increases even further to 39% for papers with RCR>10).

A similar point exists for World J Surg, where 8 of the 10 top ranked citation elite manuscripts relate to guidelines for care (with 6 stating so explicitly in the title, and/or being reviews and/or meta-analyses). This is important scientific literature, which is being recognized and impactful (to patients) beyond citations (and does not require publication in a high-impact journal for being recognized). Similarly, care guidelines are usually authored by accomplished scientists and physicians, and differ from other types of scientific literature. For instance, annual medical society meetings can host open sessions to discuss specific guidelines and invite community feedback. I have also observed authors of clinical guidelines presenting this (honorable) achievement prominently in their academic C.V.s, suggesting that biomedical scientists can recognize contribution to guidelines as important work.

These examples also all run against the simplifying presentation (without further evidence) of journal-level metrics in the context of "recall of the biomedical community's chosen measure of meritorious papers", and the authors' appeal that "we should also recognize papers in lower-tier journals that were recognized by practitioners in the field and cited equally well". The data points highlighted by the authors in Figure 1 demonstrate that the biomedical community already recognizes meritorious papers outside of high impact journals.

*- We agree that, in principle, highly cited reviews could skew our results. It was for this reason that reviews were excluded from our analysis. This has been clarified throughout the manuscript, and especially in the Methods.*

*- Regarding alternative measures of recognition, we also believe that this should be taken into consideration (i.e. contributing to practice guidelines). Other article-level metrics outside the scope of the measures presented here would be suitable for this kind of analysis (see our prior work Hutchins et al. 2019 Predicting translational progress in biomedical research PLOS Biology). These are indeed valuable contributions to article-level assessment, and we envision a broad toolbox of article-level measures that can answer a variety of questions, including but not limited to those presented in the present work.*

*- Regarding the recognition through citations, we agree that in this sense, the community is recognizing influential work, and this is the basis for our argumentation. However, the disconnect is that such values are rarely visible in hiring, promotion, tenure, and grant awards. Instead, CVs (including ScienCV, used in many federal grant applications) prominently display journal venue while leaving article level recognition through citations largely invisible. We have added additional discussion, including the caveat that not all researchers may share the sensibilities described in our manuscript, throughout the introduction and discussion.*

To summarize this brief investigation, the catchy title "… you do not receive enough recognition …" and likewise statements in the manuscript invite strong opinions that could be wrong given the additional nuances in the recognition of scientific work that lie outside of citations. I beg to hypothesize that most potential readers that would recognize themselves as part of the scientists that do not receive enough recognition would not fall into the category of authors behind above data points. Nor would I expect them to assume that the most pronounced differences between journal-level and article-level differences are driven by such (identifiable) cases (and article types outside of the scope of Nature, Science, Cell, see below). Seeing the online attention already gathered by the preprint reaffirms this concern.

*- We have toned down the language throughout the manuscript to address this concern. However, the current version of the title (changed from our original preprint) was suggested by the editor, and we will defer to his judgement on this matter.*

Further related to the interpretation of citations, Arabi et al. imply that journal impact factors are primarily used as a readout of influence. However, a recent survey by McKierna et al, eLife 2019 suggest that only 40% of institutions that consider journal impact factor do so for "impact, importance, or significance". Instead quality (63%) is the primary motivation.

*- As mentioned above, we have expanded our discussion of these matters through the introduction and discussion.*

Similarly, I suspect the portrait of Arabi et al. on "Prestigious journals" to represent only a partial view.  Rather than "pursue reputation of exclusivity by rejecting large quantitates", Nature's editorial criteria seek original scientific research of outstanding importance that has conclusions of broad interdisciplinary readership. It could be argued that none of the data points seen in my above exploration of Figure 1 fits these criteria, or fit the chosen scope of specialization by this journal. Similarly, the authors imply that journals prioritize citations, which not only runs against above stated criteria, but also anecdotal views held by some other scientists. For instance, my PhD mentor liked Nature and Cell as in his view they would recognize quality and publish important creative work independent of the size of the subfield, and thus expected citations. In the absence of data to back their assumptions, I thus believe that several assumptions of the authors may
either be wrong and/or are not ubiquitously shared among all biomedical scholars.

*- We agree that data on editorial decision making, including selection of reviewers, are sparse, especially among high profile journals. We have toned down language imputing motivations of editorial decision-making throughout the manuscript, especially in the abstract. On a side note, it would be fantastic to be able to conduct a comprehensive analysis of the decision-making process including rejected papers and reviewer selection, but such data are generally not made available to the research community. Likewise, grant decision-making data from federal funders including rejected applications are unavailable to the community to analyze, but would make a valuable contribution to the literature.*

A technical detail that is presently difficult to gauge, but could affect some conclusions of the present manuscript is the presence of purchased citations and citation rings. It however also, irrespective of the present scope, raises the point of the authors suggesting a metric that could possibly be gamed easier, when compared to journal-level metrics. In own bibliometric research around scientific fraud, I have sometimes crossed atypical citation patterns, and they should present as high RCR (as often yielding citations from other fields) in lower-tier journals. If, for instance, one considers Oncotarget, a journal now de-indexed by MEDLINE and WoS, almost 900 articles after 2010 have RCR >3.4 and would thus be considered as part of the citation elite. Most reviewers or hiring committees would not have the resources or tools (or time) to distinguish between legit membership in the citation elite and purchased membership.

*- We agree with this concern. However, we are enthusiastic about recent research efforts to identify such patterns of activity through abnormalities in the citation graph. This is similar to the methodology Google used to reduce the influence of link farms in its search engine. Gaming of RCR in particular is discussed in detail in Hutchins et al., 2016.*

Another point on citations, is that the authors compare article-level against journal-level, but their comparison contains two variables. One is article-level vs. journal level. However, this also coincides with citations vs RCR. For improved interpretability it would be good if authors could thus control for the latter, and for all key figures include an analysis where article-level is also only based on citations.

*- Reviewer 2 also raised this concern, and we have replicated our analysis using raw citations per year rather than RCR, and there is extensive supplemental data that now addresses this question. While the identity of which articles are flagged as citation elites changes due to the lack of field-normalization, we observe strikingly similar results.*

Aside from the two major sets of methodological remarks:

The manuscript focuses on what might be perceived as "false negatives", papers that belong to the citation elite but not journal elite. To better understand the described effects, it would be helpful to have some analysis that shows how many "false positive" articles would need to be read if extending the number of journals to include citation elite papers outside of the journal elite. The authors interpret the limited number of publications in high-impact journals as "artificial scarcity of recognition enforced by a journal prestige system" but do not acknowledge that Science and Nature (in particular) predate the usage of citation metrics, and that one of the traditional roles of journals is to convey relevant science to specific target audiences (with finite time for reading). Nor that several of these journals predate the electronic distribution of research articles and still appear in print. Related to this, not all highly cited papers constitute a worthwhile reading
for a broad biomedical readership (e.g.: see those data-points in Figure 1 that related to guidelines of patient care around surgeries).

*- We have conducted this analysis and included it in the revised manuscript. The impact factor would have to be relaxed to 3.0 to achieve parity between journal- and citation-elites, as reported in the results.*

The linking of papers to authors seems to be done via NIH ExPORTER. However, scientists - particularly trainees - can publish papers without being a principal investigator on an NIH grant. This could possibly skew some analysis. Inversely, some papers may cite NIH grants without the principal investigator of the latter serving as an author (for instance, in a center-type of grant, PI do not necessarily fulfill COPE authorship criteria). This could possibly also skew some analysis.

*- Apologies for any miscommunication. We have clarified that linking was done with self-reported MyNCBI profiles, which are often used to populate publications in ScienCV documents for grant applications. We agree that grant-level linking would be too imprecise for this kind of analysis.*

The performance of gender/ethnicity interference algorithms does not seem provided or validated.

*- We have now cited the peer reviewed validation study that supported the publication of the WRU package.*

The scatter plot visualizations do not directly support their usage in the text, which speaks about fraction of authors. Though the scatter plots visually are consistent with the statement of the text on there being more authors with more papers in the citation elite rather than journal elite (and thus may be considered as part of the citation elite of authors), there is no colorbar. Further, even if there was a colorbar, integrating over the colors of all data points would not be humanly possible.

*- This is correct. The visuals of citation density were conducted through transparency scales rather than color. This is now made clear in the figure legend. Instead, we color coded the pie charts to provide an alternative view for readers if color coding is preferred.*

The manuscript emphasizes the (anticipated) perspective of scientists. For instance, the authors cite one opinion piece to claim that "Many researchers in the biomedical community feel that they are unfairly judged in research assessment; that they do not receive enough credit given the influence on the research community of their published work". This statement could be read to imply that research assessment should primarily consider researchers. However, it could be also argued that research assessment - particularly by institutions - should prioritize the benefits toward society.

*- We have included additional discussion of the growing trend toward societal benefits in the discussion in response to this point, as well as the use of impact factors in decision-making as suggested by the reviewer.*


Reviewer #2:
[identifies himself as Ludo Waltman]

My comments can be found at <https://doi.org/10.21428/7ccec04a.262e56ad>


PASTED IN (unformatted):

This paper compares the assessment of researchers based on journal-level citation metrics (impact factor) to researcher assessment based on article-level citation metrics (RCR). The authors conclude that “the majority of researchers would receive more consideration using article-level compared to journal-level metrics”.

Below I offer a number of comments on the paper.

“One sensibility that often resonates with members of the scientific community that if we recognize papers during personnel advancement based in large part on the citation rate of their venue, we should also recognize papers cited equally well even if appearing in less prestigious venues”: The authors are right that arguments like this one are often made. However, it is important to acknowledge that there is disagreement about these arguments. See for instance my work on this topic: <https://doi.org/10.12688/f1000research.23418.2>.

*- This was also a concern of Reviewer 1. We have revised the manuscript to acknowledge this disagreement, and cited this work.*

The authors use the RCR metric. In my view this metric has significant problems: <https://www.cwts.nl/blog?article=n-q2u294>. I believe it would be better to use straightforward citation counts instead of RCR. Impact factors are also based on straightforward citation counts, so this would result in a more meaningful comparison between journal-level and article-level metrics.

*- I acknowledge that the reviewer and I have long-standing theoretical disagreements about RCR. Let me begin by saying that we have conducted the requested additional analysis, and extensive data show our conclusions hold when looking at raw citations (Supplemental Figure 2 and Supplemental Tables 10-17), although of course the identity of citation elite papers changes without field-normalization.*

*- Separately, the reviewer may believe that the theoretical issue posed in the cited blog post were ignored at the time of publication of the RCR metric. On the contrary, these concerns were taken very seriously and we quantified the extent to which that scenario occurs (very rarely, Hutchins et al. 2016, Figure 6). Reviewers had full access to these results. These data were contemporaneously added into a preprint update and the subsequent peer-reviewed publication.*

*- Regarding transparency, another issue raised in this blog post, the code and data underlying the RCR paper have been available on GitHub since the paper’s publication. In addition, open source iCite database snapshots are posted on Figshare and updated on a monthly cadence, and all citation edges used are publicly available in the NIH Open Citation Collection. Finally, regarding the complexity of implementation, the RCR metric methodology was reproduced within months of publication in academia, and bibliometric providers such as Elsevier, Digital Science, and Microsoft Academic each had each independently generated their own internal implementations. This computation is far less complex and far more transparent than the transformer models commonly used in the science-of-science community today.*

“The fraction of scientists that have a higher number of Citation Elite papers vs. Journal Elite papers is nearly an order of magnitude higher (Figure 2). This suggests a substantial improvement to recognition for a large segment of the biomedical research workforce by including article-level indicators as a way of recognizing research.”: I find this conclusion problematic. The authors define citation elite papers in such a way that there are many more citation elite papers than journal elite papers. It is to be expected that this then results in most researchers having more citation elite papers than journal elite papers. This is not an empirical finding but a consequence of the way the authors define citation elite papers. In the approach taken by the authors, “articles with an RCR higher than the median of those published in journals above the impact factor thresholds were labeled as article-level ‘Citation Elites’”. My suggestion is to take a different approach, namely
to choose the RCR threshold in such a way that the number of citation elite papers equals the number of journal elite papers. In my view this is necessary for a meaningful comparison between citation elite papers and journal elite papers.

*- We agree that this is important to investigate. We have conducted this analysis as requested, and found that it carries its own limitations. As now reported in the results, the impact factor threshold would have to be lowered to 3 in order to achieve parity between citation elite papers and journal elite papers. While this was a good suggestion to investigate, it is not responsive to the posed questions about recognition in the context of journals that could plausibly be prestigious enough to move the needle on decision-making. For example, megajournals could be considered ‘elite’ under this definition, a designation that would not resonate with the scientific community in any meaningful sense.*

*- The reviewer is correct that our definition of citation elites is not without limitations. The theoretical upper bounds are different for precision (when a paper is flagged as a journal elite, it is also found to be a citation elite) vs recall (when a paper is flagged as a citation elite, it is also found to be a journal elite). The theoretical upper bound for recall under this definition is 100% (all citation elites are found in journals above the specified impact factor threshold). The theoretical upper bound for precision is by definition 50%, since the median RCR or article citation rate is used for defining a citation rate threshold. As precision did not answer any of the questions posed in the manuscript, we did not note this in the Methods (although this inference should be straightforward). However, in the interests of transparency, we have included this limitation in that section.*

“Reexamination under strict zero-sum conditions”: I read this section multiple times, but I am afraid I don’t understand it. The explanation of the zero-sum framework needs improvement and further elaboration.

*- This section has been rewritten to make it more accessible.*
