# Supplementary material for: Most researchers would receive more recognition if assessed by article-level metrics than by journal-level metrics
Source: PLoS Biol. 2025 Dec 16;23(12):e3003532. doi: 10.1371/journal.pbio.3003532 (PMC12707641; doi:10.1371/journal.pbio.3003532)
Supplement: Attachment — Submitted filename: REVIEWERS_R2R.docx [file pbio.3003532.s012.docx]

REVIEWERS' COMMENTS:


Reviewer #1:

Reviewer #2:
[identifies himself as Ludo Waltman]

Comments can be found at <https://doi.org/10.5281/zenodo.17335296>

This is an interesting paper presenting a large-scale comparison between the use of journal-level and article-level citation indicators for assessing biomedical researchers.
*- Thank you for the kind comment.*

While the empirical findings reported by the authors are of significant interest, I disagree with the interpretation the authors give to their findings. In their interpretation of Figure 2, the authors conclude that their results suggest “a substantial improvement in recognition for a large segment of the biomedical research workforce by including article-level indicators as a way of recognizing research”. I disagree with this interpretation because in most contexts, in particular hiring, promotion, and funding allocation, researchers essentially find themselves in a zero-sum setting. If one researcher gets more recognition (e.g., is more likely to be hired, promoted, or funded), this implies that some other researcher will get less recognition (e.g., will be less likely to be hired, promoted, or funded).

In the final subsection in the Results section, the authors recognize that researchers often find themselves in zero-sum settings. Surprisingly, however, the authors claim that even in a zero-sum setting there are “large differences between how many authors are favored using article level metrics rather than journal level metrics”. This is an odd conclusion. In a zero-sum setting, the number of authors favored using one indicator must by definition be equal to the number of authors favored using some other indicator.

This odd conclusion turns out to follow from the specific statistical approach the authors take (without explaining it in full detail) to convert raw indicator scores into percentile ranks, as illustrated in Figure 4. According to the authors, after converting raw indicator scores into percentile ranks, there are still many more researchers who receive recognition using article-level indicators than researchers who received recognition using journal-level indicators. However, this is an artefact of the statistical approach taken by the author. In the real world, the number of opportunities to be hired, to be promoted, or to be funded is fixed, so in the real world these settings are of a truly zero-sum nature. If one researcher receives more recognition and is therefore more likely to be hired, promoted, or funded, there must be some other researcher who receives less recognition and is less likely to be hired, promoted, or funded. The use of article-level indicators instead of journal-level indicators will not result in an increase in the number of researchers who can be offered a job or a promotion, or who can be awarded funding.

There are important reasons to criticize excessive reliance of journal-level indicators, but the argument presented by the authors is not convincing. The empirical insights provided by the authors are valuable, but the authors need to rethink the interpretation they give to their findings.

*- We have added two responses to these ideas in the Discussion, which we feel strengthens the manuscript and hope the readers find interesting:*

*- “It might be argued that improvements in recognition are impossible under the zero-sum conditions of the biomedical research enterprise, since the number of positions or honors is fixed, any gain for one researcher necessarily entails a loss for another. There are two rejoinders to this line of argumentation, one direct and one indirect. The first is that additional information can still improve the fairness and accuracy of recognition, even in zero sum systems. Researchers who have no Journal Elite papers but do have Citation Elite papers would benefit from the acknowledgement that their work, despite being published in less elite journals, has had comparable influence to that in top-tier venues.* ***This may indeed improve their relative ranking and decrease that of others****.* ***But for it to be true that no improvement in recognition has been achieved, one would have to assert that even highly cited papers in lower-tier journals are undeserving of recognition solely because of their publication venue****.* ***The existence of a zero-sum system does not obviate the reduction of uncertainty in decision-making that an additional, or even alternative, information source provides****. In fact, it may be in zero-sum hypercompetitive conditions that such improvements to the decision-making process are most needed.”*

*- “The second rejoinder to this argument is that while it may be true that the use of article-level measures does not increase the number of positions at a given moment, they can contribute to expanding opportunities over time. We have powerful examples of article-level metrics contributing to growth in overall biomedical research funding by engendering trust with legislators who set the budget. Former NIH Director Francis Collins used article-level metrics as the building blocks of bird’s-eye-view analyses of the biomedical funding landscape to continuously evaluate the research portfolio, make difficult policy decisions, and demonstrate effectiveness to the United States legislature during appropriations meetings: “These types of tools provide NIH with the evidence base it needs to make funding decisions that promote an efficient and impactful biomedical research portfolio.” Initiatives to increase the overall research budget, as seen for NIH from 2016 to the pandemic, are more credible when accompanied by rigorous quantification that starts from a granular level and aggregates up to a landscape view. Demonstrating that scientists can quantify and even forecast outcomes of the research enterprise beyond what peer review alone provides represents an important step towards sustaining and expanding the biomedical research enterprise. Because trust in science has declined since the public health policies during the pandemic, every source of added confidence is needed to foster a growing research enterprise.”*

*- In addition, we have documented in the Methods the percent_rank function used, from the commonly used dplyr R package.*

Finally, a minor comment I have relates to the RCR indicator used by the authors to measure the citation impact of an article. There has been some debate about this indicator. See the following paper by Janssens and colleagues: https://doi.org/10.1371/journal.pbio.2002536. And see also the following blog post by myself: https://www.cwts.nl/blog?article=n-q2u294. In my view, the authors should inform readers that there are different perspectives on the pros and cons of the RCR indicator.

*- We have acknowledged that RCR has stirred debate and cited Janssens as well as a Nature piece documenting the debate.*
